# Building a Community Partnership for the Development of Health Ministries Within the African American Community: The Triad Pastors Network

**DOI:** 10.1007/s10900-023-01315-4

**Published:** 2024-01-24

**Authors:** TanYa M. Gwathmey, K. Lamonte Williams, Allison Caban-Holt, Takiyah D. Starks, Capri G. Foy, Allison Mathews, Goldie S. Byrd

**Affiliations:** 1 Maya Angelou Center for Health Equity, Wake Forest University School of Medicine, Winston-Salem, NC USA; 2 Hypertension and Vascular Research Center, Department of Surgery, Wake Forest University School of Medicine, Biotech Place – 575 N. Patterson Avenue, Suite #340, Winston-Salem, NC 27101 USA; 3 Division of Public Health Sciences, Department of Social Sciences and Health Policy, Wake Forest University School of Medicine, Winston-Salem, NC USA; 4 COMPASS Initiative Faith Coordinating Center, Wake Forest University School of Divinity, Winston-Salem, NC USA

**Keywords:** Community engagement, Faith-based organizations, Health equity, Health disparities, African Americans, Health literacy

## Abstract

African Americans continue to have worse health outcomes despite attempts to reduce health disparities. This is due, in part, to inadequate access to healthcare, but also to the health care and medical mistrust experienced by communities of color. Churches and worship centers have historically served as cultural centers of trusted resources for educational, financial, and health information within African American communities and a growing number of collaborations have developed between academic institutions and community/faith entities. Herein, we describe the infrastructure of a true and sustainable partnership developed with > 100 prominent faith leaders within the Piedmont Triad region of North Carolina for the purpose of developing or expanding existing health ministries within houses of worship, to improve health literacy and overall health long-term. The Triad Pastors Network is an asset-based partnership between the Maya Angelou Center for Health Equity at Wake Forest University School of Medicine and faith leaders in the Piedmont Triad region of North Carolina that was created under the guiding principles of community engagement to improve health equity and decrease health disparities experienced by African American communities. A partnership in which co-equality and shared governance are the core of the framework provides an effective means of achieving health-related goals in a productive and efficient manner. Faith-based partnerships are reliable approaches for improving the health literacy needed to address health disparities and inequities in communities of color.

## Introduction

Despite many attempts to reduce health disparities, African American communities continue to have worse health outcomes. While there have been significant advances made towards controlling cardiovascular disease morbidity and mortality in recent decades, African Americans continue to experience a markedly elevated burden of cardiovascular disease [1–3], type 2 diabetes [4], Alzheimer’s disease and related dementia [5, 6] and cancer [7]. In the context of the COVID-19 pandemic, these health disparities were magnified as African Americans were disproportionately represented in morbidity and mortality rates [8, 9].

In addition to inadequate access to healthcare and related resources, one of the primary factors associated with impaired progress towards reducing health disparities in African Americans is lack of trust in the healthcare and scientific communities, attributed to a history of scientific misconduct by these institutions [10–12]. Moreover, even in the current climate of focused efforts to mitigate health disparities in communities of color, few studies adequately address the needs of African Americans or seek their involvement in the development of research studies.

Churches, worship centers and other faith-based organizations within the African American/Black community serve as highly-influential institutions and cultural centers of information as well as agents of change [13]; hence, they are well-positioned to provide educational, financial, social, and health-related resources to their congregants and their surrounding communities. Consequently, health promotion through churches and worship centers has received growing interest as a means to reduce health disparities [14], including focused areas such as mental health [15, 16], cardiovascular and cardiometabolic diseases [17–25], cancer [26–28] and HIV [29–33]. Moreover, strategic tools have been developed to assess the readiness of African American churches to engage in health promotion programming [34–36].

Indeed, as described above, a growing number of studies have reported initiatives where academic researchers have sought collaborations with churches or faith-based entities for participation in research studies of various types. Johnson et al., 2020 describe an alliance initiated by faith-based leaders with academic researchers to participate in patient-centered health research initiatives; however, the present work describes a partnership developed with academic researchers and healthcare providers together with pastors and faith leaders to establish or expand existing health ministries within local churches, for the purpose of improving health and health literacy through education, equipping and empowerment. Herein, we describe the development and infrastructure of the Triad Pastors Network (TPN), an interfaith association of over 100 + churches and faith-based organizations within the Piedmont-Triad region of North Carolina, and the Maya Angelou Center for Health Equity (MACHE) at Wake Forest University School of Medicine. The goal of this endeavor is to improve health equity and reduce health disparities in African American communities, while providing pastoral care and support to faith leaders, and their congregants.

## Approach

All activities of the Triad Pastors Network (hereafter referred to as the *TPN* or *Network*) have been approved by the Wake Forest University School of Medicine Institutional Review Board (IRB# 00068318).

The TPN was conceptualized during 2018 and the following strategic steps were outlined to ensure its success towards meeting the stated goals and objectives.

### Step 1: Building a Team for Faith Engagement

To support the efforts of the faculty and staff of MACHE towards developing the TPN, two additional team members with long-standing histories of faith and community engagement were recruited to join the Coordinating Team. Each of these individuals brought the experience of pastoring a local church for > 10 years and currently serve as faith leaders within their respective houses of worship. Additionally, one of the faith leaders is a formally trained scientist and is engaged in biomedical sciences research focused on health disparities.

**Fig. 1:**
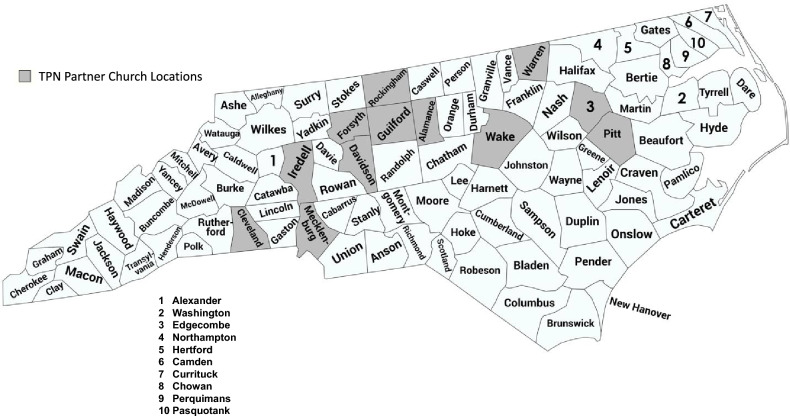
Mapping of participating churches in the Triad Pastors Network across the state of North Carolina

Members of the TPN (pastors and faith leaders across the Piedmont Triad region) were solicited for partnership through various networks and relations of the MACHE Coordinating Team throughout the year of 2019. A mapping of the location of participating churches can be seen in Fig. 1. While the greatest number of participating churches are localized to Forsyth and Guildford counties, 50% and 22%, respectively, involvement of churches across central North Carolina is illustrated in Table 1.

**Table 1:** Distribution of participating TPN churches

North Carolina geographic regions by county	Percentage (%)
Triad—Forsyth	50
Triad—Guilford	22
Triad—Davidson	6
Triangle—Wake	4
Triad—Alamance	3
Charlotte MSA—Iredell	3
Charlotte MSA—Mecklenburg	3
Triad—Rockingham	3
Triangle—Warren	3
Western—Cleveland	3

Data is presented by geographic regions within the state of North Carolina

*MSA* metropolitan statistical area

Candidate partner churches were asked to attend a *listening tour*, where the credentials of the leader and the team were highlighted, and the concepts around health promotion and creation/expansion of health ministries within local churches were presented. Additionally, each faith leader was asked to share areas of health that were of particular importance to their congregation and community. After consenting to participate in the partnership, each faith leader was asked to complete a *Memorandum of Understanding*, which described the nature of the partnership and expressed the expectations of all stakeholders involved.

While church attendance is often a dynamic variable that increases or decreases with time and various socio-economic factors, the average church membership within the U.S. is 100 individuals [38], providing a potential reach of 10, 000 + persons from the > 100 participating churches as a collective audience of the TPN. However, several of the TPN pastors and faith leaders serve as regional and/or national leaders of their denominations or reformations, with multiple churches under their purview. For example, one TPN member has ~ 50 distinct churches under his direct leadership, thus health information disseminated to TPN members has the capacity to impact tens of thousands across multiple regions.

### Step 2: Assessing Faith Leaders’ Desired Objectives and Areas of Health Emphasis

To ensure that the partnership reflected the mutual goals of the Faith Leaders as well as that of MACHE, each faith leader was asked to complete a survey indicating their top health priorities (reaffirming what was shared during the listening tours), as well as an assessment of assets that could benefit the members of the Network towards health promotion events (e.g. church gardens, walking trails, fellowship halls and kitchens). A mapping of these assets was included among the listing of assets provided through the Wake Forest University School of Medicine, which included access to affiliate faculty with expertise in various areas of health and disease, in addition to material resources.

### Step 3: Developing the Mission, Vision and Goals

After evaluating the collective input of each TPN partner member, the *mission*, *vision*, and *goals* of the Network were created. A Pastors’ Retreat was held in December 2019 for the purpose of establishing the mission, vision and goals among the partner members. Feedback provided from the faith leaders during the listening tours and results of the asset mapping were shared among participants. Breakout sessions using small group format were held during the retreat to brainstorm approaches to address key health topics raised by faith leaders. This unifying event, attended by over 120 faith leaders and community activists, served to coalesce the TPN members towards the urgency of creating health ministries to improve the health of their local congregations and communities.

### Step 4: Establishing an Ecclesiastical Council Advisory Board

**Fig. 2:**
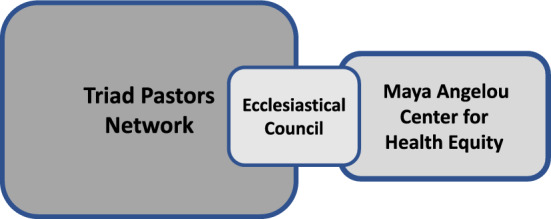
Leadership Structure of the Triad Pastors Network. The Ecclesiastical Council is comprised of a subset of faith leaders from the TPN at large, as well as individuals from the MACHE Coordinating Team

In an effort to develop an advisory board that would guide the initiatives and activities of the Network, and to ensure the protected interests of all stakeholders in the partnership, an Ecclesiastical Council was created. The Ecclesiastical Council was comprised of twenty-six community faith leaders who are members of the TPN body at large, including two individuals who do not currently pastor churches, but provide leadership of large community organizations; two faith leaders that are part of the MACHE Coordinating Team and also serve as local pastors; and the Executive Director of MACHE, thus providing representation from all stakeholder partners (see Fig. 2). The faith leader members were selected to represent each of the primary demographics of the TPN at large, and consist of both male and female faith leaders, individuals who pastor churches in rural, suburban and urban settings, and those who oversee large (> 250 members), medium (100–250 members) and small (< 100 members) congregations (see Table 2). The churches that comprise the Council similarly have broad diversity in geographic locations and include the counties of Forsyth, Alamance, Mecklenburg, Edgecombe, Guildford, Iredell, Warren, Davidson, Rockingham, Onslow, Wake, Cleveland and Pitt. Moreover, the composition of the Council is comprised of 90% African Americans, and 10% who identify by more than one race.

**Table 2:** Composition of the TPN Ecclesiastical Council. The Council is comprised of 26 faith leaders from the TPN and 3 members of the Maya Angelou Center for Health Equity Coordinating Team

	N = number of participants	Percentage (%)
Total number of Ecclesiastical Council members	29	
Females	6	21
Males	23	79
Geographic location of churches/worship centers		
Rural	4	15
Suburban	6	23
Urban	16	62
Size of congregation		
Large (> 250 members)	9	31
Medium (100 – 250 members)	9	31
Small (< 100 members)	8	28
Does not currently oversee a church/worship center	3	10

A set of *Terms of Engagement* was created to maintain appropriate functioning of the Ecclesiastical Council by designating the role and responsibilities of the Council, a code of conduct, and appointment terms for its members. The Terms of Engagement were voted on and approved by the Council members for implementation, as are all initiatives and activities under consideration for adoption by the TPN.

### Step 5: Establishing an Approach to Health Promotion and Implementation of Health Ministries

**Fig. 3:**
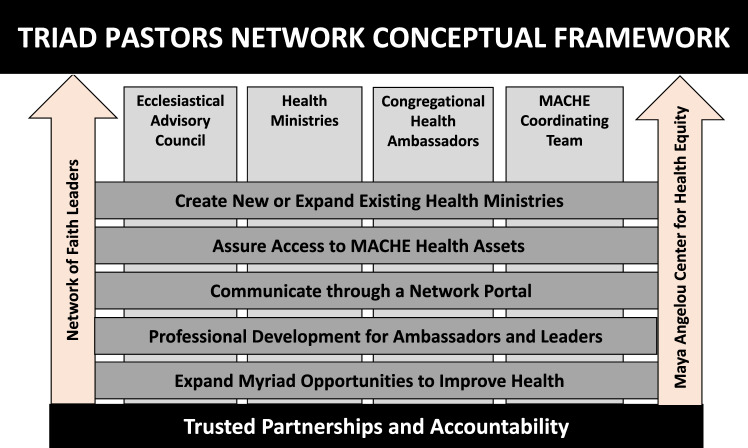
The Triad Pastors Network Conceptual Framework. *MACHE* – Maya Angelou Center for Health Equity

Following the creation of the Ecclesiastical Council Advisory Board, the mission, vision and goals that were originally developed during the Pastor’s Retreat were re-assessed to ensure that they remained in alignment with the values of the TPN. Further, approaches to promotion of health topics and implementation (or expansion) of health ministries within the TPN member churches were detailed to reflect the diversity of culture within partner members churches. This approach included the identification and appointment of a *congregational health ambassador (CHA)* by the faith leader, to serve as a liaison for disseminating health information from MACHE to local churches, and ensuring access to MACHE health assets. A Network portal made available to all stakeholders serves as an online data bank of health resources within MACHE, partner churches and the community. In addition to education on select health topics, both the CHA and Faith Leader receive education on various professional development skills and activities (including leadership and organizational skills, project management and networking) that could potentially enhance the administrative structure of the ministry (see Fig. 3).

## Results

**Fig. 4:**
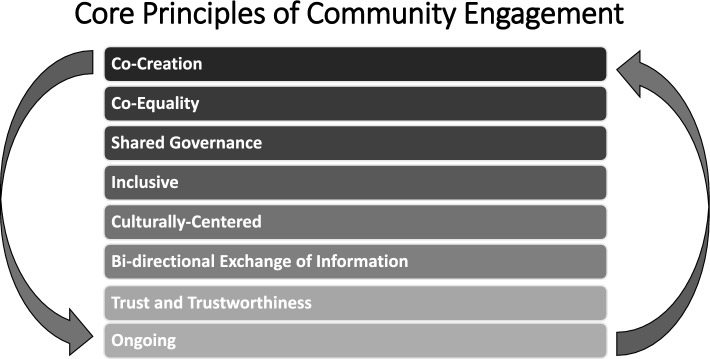
Core Principles of Community Engagement, as adopted from Centers for Disease Control and Prevention (CDC). [39]

Our approach to creating a successful community partnership employs each of the *core principles of community engagement* (see Fig. 4).

### Co-Creation

The TPN was co-created by a team of academic research investigators with a background in health equity and health disparities research, and an interfaith group of community pastors and faith leaders. The academic investigative team includes two pastors/faith leaders to provide culturally appropriate context for interactions with the Faith Community, and expertise in community engagement.

### Shared Governance

The TPN operates through the shared governance of the MACHE Coordinating Team and the Ecclesiastical Council, a subset of the faith leaders that represent the diverse array of churches that comprise the TPN, and serve as an advisory board for the activities of the Network. The Ecclesiastical Council is guided by a set of *Terms of Engagement* that were developed in collaboration with all stakeholders, and each member of the Ecclesiastical Council and TPN at large is required to complete a Memorandum of Understanding that expressly states the expectations for all parties involved in the Network.

All initiatives are first proposed to the Ecclesiastical Council members to gain approval by majority vote before implementation. This approach creates an avenue for all stakeholders to provide input before an initiative is implemented, and to ensure that the best interests of all partners are considered.

### Co-Equality

The governance structure described above is set to ensure that co-equality exists within the partnership. Common occurrences that are frequently observed in collaborations between academic institutions and community partners are (1) a true partnership does not exist; such that both parties do not have equality in the partnership, and it is often the community partner that is subordinate to the academic institution; (2) the goals and objectives are not commonly conceived by all stakeholders, often leaving the community partner(s) in a position of consequential rather than deliberate benefit. Often, these benefits are only experienced provided the goals of the academic institution are met; and (3) the needs of the community partner(s) are not determined by the community partner(s), but rather assumed by the academic institution.

Our approach during the creation of this partnership included listening tours where faith leaders were provided opportunities to express their key interests and areas of health promotion that were of greatest importance to their congregation and community. Further, the approaches pursued to implement health promotion events and creation/expansion of health ministries were developed and approved by the faith leaders. Importantly, the mission, vision and goals of the Network were reassessed to ensure that they represented the perspectives of the faith leaders and not the MACHE Coordinating Team alone.

### Bi-directional Exchange of Information

It is our policy to provide timely information to our partners concerning all TPN-related activities. In particular, when surveys are administered to assess various aspects of health within the faith leaders’ congregations, the results of those surveys are quickly returned to the leaders and presented in a manner that is conducive for their use. Additionally, the results are discussed with the members of the Network to develop strategies to address any areas of concern.

### Trust and Trustworthiness

Building trust is essential for partnerships that involve community stakeholders and researchers, particularly for vulnerable populations and those who have experienced medical mistrust. Foremost in the development of the TPN was the establishment of trust between all stakeholders. We aimed to achieve trust by ensuring complete transparency in all activities and in all communication. Furthermore, we have established an agreement with our partners that we will not provide access to the Network to other research investigators without their expressed consent to do so.

### Culturally Centered

As our overarching goal is to improve health equity and reduce health disparities in African American communities, our partnership includes highly influential faith leaders within local African American communities. Moreover, health educational sessions are carried out by MACHE Affiliate faculty who have cultural sensitivity and culturally appropriate training to share health information to audiences provided by our faith partners. We deliberately select session facilitators with whom members of the audience can relate and feel free in their communication to ask relevant questions. Additionally, we aim to develop culturally appropriate materials to relay important health information so that the messaging is not lost in its delivery or presentation.

### Inclusive

Recognizing that faith communities are not homogenous groups of people, and that considerable diversity exists within our Network, our community faith leaders oversee a diverse array of houses of worship that span the gamut of urban to rural churches; smaller (< 50 members) to larger congregations (> 4000 members); and vary in the average age of attendees, as well as approaches to worship (traditional versus contemporary). We adopt approaches to health promotion that will reach audiences on each end of the spectrum. These approaches are guided by our Ecclesiastical Council and tailored to be effective in their respective environments.

### Multi-knowledge

Early in the development of the TPN partnership, we learned that there was great knowledge to be gleaned from our partners, and that this information was as valuable as the health information we intended to share. Collectively, our partners have decades of experience with effective modes of community engagement, and are quickly able to identify approaches of engagement that are ineffective within African American communities, particularly faith communities, and communities of various ages. Moreover, our partners assist us with crafting relevant outreach tools and methodology, while we share with them appropriate applications of health information.

### Equitably Financed

While MACHE provides its faith leader partner churches with access to a rich bank of health resources through the Wake Forest University School of Medicine, the premise of the TPN is an asset—based partnership that reflects contributions by the faith partners as well. This shared approach to supporting the activities of the Network allows mutual benefit by all of the stakeholders, and helps to preclude dominance by a single stakeholder that is attributed to their wealth of support.

### Ongoing

One of our long-term objectives is to ensure sustainability of the Network and long-term maintenance of health ministries within the local churches. This objective exists to ensure that African American community members are empowered and equipped to address their health concerns, in an effort to achieve health equity and to reduce health disparities. Frequently, academic-community partnerships are severed once the aims of the study are completed by the academic entity; however, the TPN partnership exists to help African American communities leverage resources and assets in order to begin prioritizing health ministries as part of the long-term components of the church.

## Conclusions

Individuals and the communities in which they reside are deeply impacted by the systems and infrastructure that drive decisions and influence their health; however, these individuals are often not included in the processes that create programs and policies designed to benefit them [39]. This is especially true for communities of color and other underserved populations. We have developed a trusted partnership with prominent faith leaders in local African American communities in order to create health ministries to be sustained long-term in efforts to improve health literacy and overall health. This partnership with the faith community was inspired to establish a trustworthy resource for health information, mitigating the medical mistrust experienced by African Americans and communities of color throughout the course of time.

Our approach to developing this partnership was guided by the core principles of community engagement, to ensure its success and that the goals and objectives of the initiative are met. The step-wise approach to developing this partnership is generalizable and could be adopted for implementation in other geographic regions of the country to broaden impact. However, as churches vary extensively in their governance structure, staffing, connections with other community organizations, and in the size and characteristics of congregations, it is important to tailor the approach to developing a health ministry to fit the culture and needs of that church.

Although there is an increasing number of academic and faith-based community collaborations emerging, the current report details the development of a true partnership that was created not for the purpose of enrolling community participants in research studies, but rather to educate, empower and equip these individuals to improve their health literacy and overall health by developing (or expanding an existing) health ministry within their church/worship center. Moreover, few published reports include partnerships with a network of this scale (> 100 churches), thereby increasing the likelihood that the initiative would cease following the disassociation of more than one church. We have described an approach that will help to ensure long-term sustainability, despite cessation of participation by a number of churches. Additionally, participation by a large number of partners helps to promote recruitment and engagement of new members on an ongoing basis, and serves to create a larger sense of community for participants, beyond that surrounding their house of worship.
